# Metabolome and transcriptome profiling unveil the mechanisms of light-induced anthocyanin synthesis in rabbiteye blueberry (*vaccinium ashei*: Reade)

**DOI:** 10.1186/s12870-022-03585-x

**Published:** 2022-04-29

**Authors:** Xiaolan Guo, Muhammad Shakeel, Delu Wang, Chunpu Qu, Shimei Yang, Shahbaz Ahmad, Zejun Song

**Affiliations:** 1 College of Forestry, Guizhou University, Guiyang, Guizhou China; 2 College of Life Sciences, Huizhou College, Huizhou, Guangdong China; 3 Department of Entomology, University of the Punjab, Lahore, Pakistan

**Keywords:** Blueberry, Flavonoids and anthocyanin accumulation, Light intensity, Metabolome and transcriptome, *VcbHLH004*

## Abstract

**Background:**

Blueberry is one of the most important fruit crops worldwide. Anthocyanin is an important secondary metabolites that affects the appearance and nutritive quality of blueberries. However, few studies have focused on the molecular mechanism underlying anthocyanin accumulation induced by light intensity in blueberries.

**Results:**

The metabolic analysis revealed that there were 134 significantly changed metabolites in the natural light compared to the control, and flavone, flavonol, and anthocyanins were the most significantly increased. Transcriptome analysis found 6 candidate genes for the anthocyanin synthesis pathway. Quantitative reverse transcription PCR (qRT-PCR) results confirmed changes in the expression levels of genes encoding metabolites involved in the flavonoid synthesis pathways. The flavonoid metabolic flux in the light intensity-treatment increased the accumulation of delphinidin-3-O-arabinoside compared to under the shading-treatment. Furthermore, we performed qRT-PCR analysis of anthocyanin biosynthesis genes and predicted that the gene of *VcF3’5’H4* may be a candidate gene for anthocyanin accumulation and is highly expressed in light intensity-treated fruit. Through the co-expression analysis of transcription factors and anthocyanin synthesis pathway genes, we found that the *VcbHLH004* gene may regulate *VcF3’5’H4*, and then we transformed *VcbHLH004* heterologously into tomato to verify its function.

**Conclusion:**

These results provide novel insights into light intensity regulation of blueberry anthocyanin accumulation and represent a valuable data set to guide future functional studies and blueberry breeding.

**Supplementary Information:**

The online version contains supplementary material available at 10.1186/s12870-022-03585-x.

## Background

Color constituents are important economic traits in some fruit and vegetables that are present due to high anthocyanin accumulation. Anthocyanin is a flavonoid that is vital for the production of fruit quality and color [1, 2]. The concentration of anthocyanin during fruit ripening is related to the soil, growing area, and climate [3, 4]. Light is a crucial environmental factor that affects anthocyanin synthesis in many plants [5]. Among them, light intensity and light quality are the most significant. The expression of structural genes and transcription factors related to anthocyanin biosynthesis can be regulated through signal transduction pathways that affect anthocyanin synthesis and its accumulation [6]. Therefore, the effects of light and other environmental factors on anthocyanin synthesis are essential to study and we need to find the mechanism of regulation for improving fruit quality.

Higher light intensity can increase anthocyanin accumulation in many plants [4, 7]. Azuma et al. (2012) showed that strong light may significantly induce anthocyanin accumulation in grape peel and simultaneously induce the high abundance expression of *DFR*, *CHS*, *CHI*, *F3H*, *MT*, *F3’5’H*, and *GT* [8]. The anthocyanin concentrations under blue and red light treatments were higher as compared to dark conditions [9, 10]. During the color transition period, UV-light alters concentrations of anthocyanin in blueberries [11]. In the pericarp, continuous illumination of blue and red light increased the concentration of anthocyanin. However, blue light enhanced the accumulation of anthocyanin more than red light [12]. The concentration of anthocyanin in grapes treated with strong light-intensity was significantly different as compared to control treatments [13]. These studies showed that light increases the accumulation of anthocyanin. In plants, the anthocyanin biosynthesis pathway is regulated by several structural genes and regulatory transcriptional factors (TFs) and plays a significant role in the production of diverse anthocyanin components [14].

Anthocyanin contents were changed under light exclusion due to a decrease in the transcriptional level of anthocyanin regulation and structural genes [15]. In grapes, Marselan (*V. vinifera* L.), shade treatment can reduce the content of anthocyanin by changing the expression of anthocyanin synthase genes (*CHS*, *CHI*, *DFR*, *F3H*, *LDOX*, *F3’5’H*) and regulatory gene expression (*VvMYB30*, *VvbHLH79*, *VvbHLH121*) [16]. Furthermore, research showed that the expression of structural genes (*LDOX*, *CHS*, *F3H*, *DFR, CHI*, *UFGT*), and regulatory genes (*MybA1*) that were exposed to strong light caused down-regulation, resulting in decreased anthocyanin content in wine grapes (*Vitis vinifera*) [17]. The molecular mechanisms of light intensity regulating fruit anthocyanin biosynthesis have been reported in pear [18], peach [19], and grape [20]. Therefore, studying the influence mechanism of light intensity on the synthesis of plant anthocyanin is an important basis for improving plant anthocyanin.

Blueberry production was estimated at 347,200 tons with a cultivated area of 66,400 hm^2^ in China [21]. Within Guizhou Province, blueberries are planted with a cultivated area of 15,000 hm^2^ and a production of 85,000 tons [21]. Anthocyanin accumulation in blueberries is closely related to the light signal. Many studies have reported the effect of light quality on blueberry anthocyanin synthesis, such as, UV-B treatment can increase the expression of anthocyanin regulatory factors *VcMYBA1*, *VcMYBPA1*, and *VcMYBC2,* enhancing the accumulation of anthocyanin in blueberries [14]. Nguyen’s research showed that UV-B promoted the expression of *VcBBX*, *VcMYB21*, and *VcR2R3 MYB* transcription factor, leading to the accumulation of anthocyanin. Recently, the accumulation mechanism of blueberry anthocyanin has been studied using light quality. However, the molecular mechanism of light intensity regulating blueberry anthocyanin synthesis has not been reported yet. In the present study, we reported the anthocyanin accumulation by metabolome and transcriptome analyses in rabbiteye blueberry ‘Pink Blue’ fruit samples treated with different light intensities in order to study the molecular mechanism of anthocyanin synthesis. This study greatly expands our understanding of the molecular mechanism of light intensity in regulating blueberry anthocyanin accumulation.

## Materials and methods

### Plant materials

Five-year-old rabbiteye blueberries (*Vaccinium ashei*: Reade) were grown at Guiyang, Guizhou, China. The planting distance was 30 × 30 cm. The growing region located at 106°27′-106°52′ East longitude and 26°11′-26°34′ North latitude. The light treatment and fruit harvesting time were March 2020 and June 2020, respectively. Multiple shading nets and illuminance metre (Shandong Rongcheng Shading Online Shop) were used to measure different levels of shading and intensity of light, respectively. Thirty blueberry plants were selected under different light treatments, including: natural light (intensity 100%: CK), light shading (intensity 75%: H), moderate shading (intensity 50%: F) and severe shading (intensity 25%; Q). The samples were frozen in liquid nitrogen and stored at − 80 °C for further pigment composition, metabolite, qPCR analyses, and RNA sequencing (RNA-Seq).

### Measurement of chlorophyll, carotenoid, total anthocyanin, and total flavonoids

The chlorophyll and carotenoid contents were determined using 80% acetone mentioned previous method [22]. For this, 1.0 g of fruit (fully ground) was taken and added 10 ml of 80% acetone for shading and extraction until the leaves completely whitened. Absorbance values were measured at wavelengths of 663 nm, 645 nm and 470 nm in a UV spectrophotometer. The formulae for calculating chlorophyll concentrations were such as: chlorophyll a = (12.72A663–2.59A645)V/(1000 m); and chlorophyll b = (22.88A645–4.67A663)V/(1000 m). The formulae for calculating carotenoid concentrations were as follow: carotenoids = (1000A470–3.27C_a_ – 104C_b_)V/(229 × 1000 m), and the results were expressed as mg g^− 1^. The formulae for total chlorophyll were as following: (8.05A663 + 20.29A645)V/(1000 m). In the formula, Ca represents the concentration of chlorophyll a; Cb represents the concentration of chlorophyll b; V is the volume of the extract; m is the fresh weight of the sample. The content of total flavonoids was determined by double-antibody one-step sandwich method-enzyme-linked immunosorbent assay (ELISA).

### Metabolomic analysis

Metabolite profiling was performed using a widely targeted metabolome method using Wuhan Metware Biotechnology Co., Ltd. (Wuhan, China) (http://www.metware.cn/). Freezing-dried fruit skin was crushed into powder using a mixer mill (MM 400, Retsch). The fruit (1 cm wide and 0.2 cm thick along the fruit lengthwise) were sampled with different light intensities, and three replicates for each of Lv and Bai. A total of 100 mg powder was extracted overnight at 4 °C with 1.0 ml 70% aqueous methanol, then centrifuged at 10, 000 g for 10 min. After that, these extracts were absorbed, filtrated, and analyzed by an LC-MS/MS system. Analytical conditions were based on the procedures as described in Zhang et al. [23]. Quantification of metabolites was carried out using a MRM method [24]. Metabolites with significant differences in content were set with thresholds of variable importance in projection (VIP) ≥1and fold change ≥2 or ≤ 0.5 [25].

### Transcriptome sequencing and enrichment analysis

Highbush blueberry (*V. corymbosum* hybrids) reference genome was used this link (http://gigadb.org/dataset/100537) [26]. Alignments were made using HISAT2 software to map the filtered reads for the reference genome [27]. The filter conditions and false discovery rate (FDR) were: |log2 (fold change)| ≥ 1, and < 0.05, respectively. The Kyoto Encyclopedia of Genes and Genomes (KEGG) and Gene Ontology (GO) enrichment analyses of the DEGs were implemented using the Cluster profiler R software packages (*p*-value < 0.05) [28]. The online database Plant TFDB was used to identify transcription factors [29]. The transcription factor and co-expression of anthocyanin synthesis pathway genes were visualized using Cytoscape [30]. The blueberry anthocyanin synthesis pathway was identified using blast and the E-value was e^− 5^ [31].

### Correlation analysis of the metabolome and transcriptome

Pearson correlation coefficients were calculated for the integration of the metabolome and transcriptome information. The mean metabolite content and mean expression of each transcript were calculated according to the metabolomic and transcriptomic data respectively, using coefficient calculation. The fold changes in each group (Shading and Lighting groups, CK/Q, CK/F, and CK/H) were calculated for both the metabolome and transcriptome data. The coefficients were calculated from log2 (fold change) of each transcript and log2 (fold change) of each metabolite using EXCEL program. Metabolome and transcriptome relationships were visualized using Cytoscape (version 3.7.2). Correlations coefficient of R2 > 0.8 and *p*-values<0.05 were selected.

### Ectopic expression of *VcbHLH004* in Micro-Tom tomato

The CDS of *VcbHLH004* was recombined into the pBWA(V)HS-ccdb-GLosgfp vector for gene transformation with the 35 S promoter and then transformed into *Agrobacterium tumefaciens* GV3101 using the floral-dip method. For half-strength Murashige and Skoog (MS) solid medium with kanamycin, T1 transgenic plants were selected. Kanamycin resistant seedlings were grown in a light incubator under a 16-h light/ 8-h dark photoperiod at 24 °C. Ripe fruit were used for anthocyanin identification, quantification, and RNA-seq analysis.

### RNA extraction 、library construction and qRT- PCR validation

Twelve libraries were constructed for transcriptome sequencing that represented four peel samples and three replicates. RNA extraction, transcriptome sequencing, and quantification were performed [21]. For library sequencing on the Illumina Hiseq platform, 150 bp paired-end reads were generated for the library. For RNA-Seq., total RNA was extracted and transcribed into cDNA using the PrimeScript™ RT Reagent Kit (TaKaRa) in accordance with the manufacturer’s instructions. qRT-PCR was performed in a Real-time PCR System (ABI QuantStudio 3, ABI) using SYBR Real Master Mix (Transgen, Beijing, China) under the following PCR thermal cycling conditions: predenaturation at 95 °C for 30 s; followed by 40 cycles of 95 °C for 15 s, 60 °C for 30 s, and 95 °C for 15 s. The sequences of the primers used are listed in Supplementary Table S1. ACT7 and GAPDH (AY123769) bilberry genes were used as the housekeeping gene. Tree biological replicates were performed for each gene, and the standard curve method was applied in statistical analysis.

## Results

### Effect of light intensity on contents of pigment in blueberry fruit

**Fig. 1:**
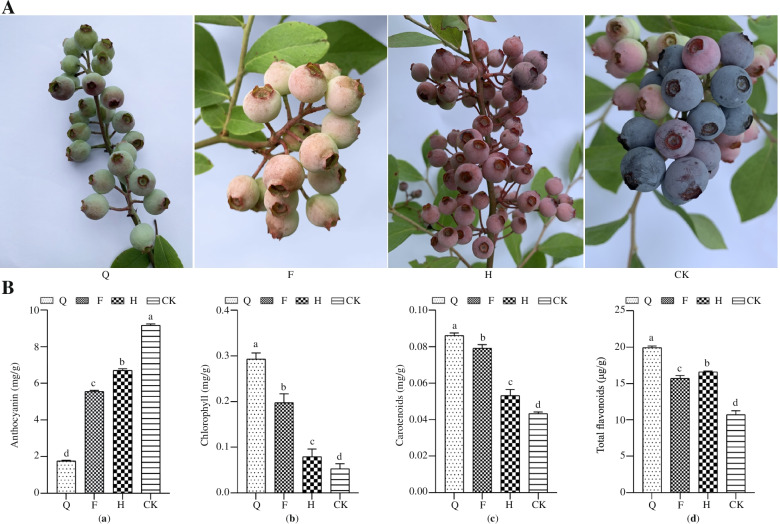
Fruit color phenotype under different light intensity (**A**); Analysis of pigment content under different light intensity (**B**). Note: Lowercase letters indicate significant differences between treatments (*P*<0.05). Note: Q: light transmittance 25%, F: light transmittance 50%, H: light transmittance 75%, CK: light transmittance 100%

Strong light significantly enhanced blueberry fruit growth and development that led towards fruit maturation and ripening (Fig. 1A). Anthocyanin content was increased due to strong and reduced the content of carotenoids, chlorophyll and total flavonoids (Fig. 1B).

### Metabolite analysis

Fruit samples were collected from the different light intensities used to measure changes in metabolite concentration associated with the peel coloration process in blueberries (Fig. 1A). We evaluated the metabolomes of the twelve samples using the widely-targeted metabolomics approach. We detected 134 compounds grouped into 11 classes (Table S1). Metabolite concentration data was used to perform hierarchical heatmap cluster analysis. All the biological replicates were grouped together representing the high-reliability of the generated metabolome data (Fig. S1). Interestingly, we observed a separation between natural light (CK) and 25% light transmittance (Q) which indicated that the metabolite spectra of the two samples were significantly different (Fig. S1).

**Fig. 2:**
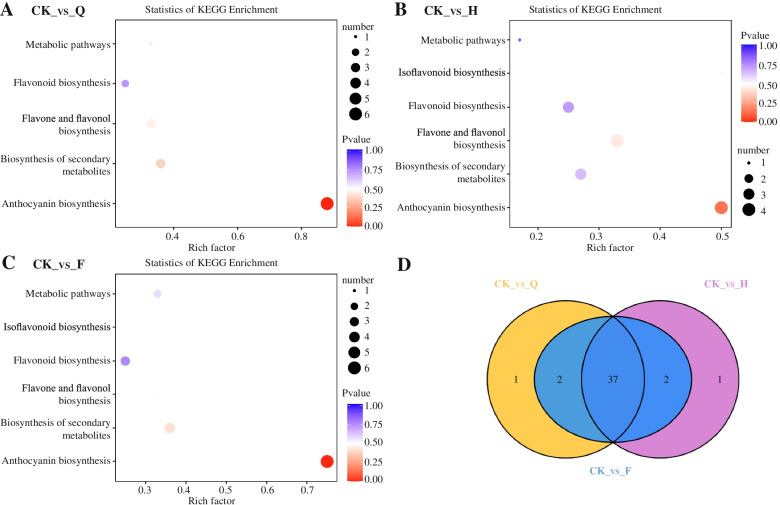
Identification and functional characteristics of differentially accumulated metabolites in fruit samples with different light intensities. Note: KEGG enrichment analysis of the DAMs between (**A**) CK-vs-H, (**B**) CK-vs-F, (**C**) CK-vs-Q,and (**D**) Venn diagram depicting the shared and specific metabolites between the three compared groups of peel samples. CK: natural light, H: light transmittance 75%, F: light transmittance 50%, Q: light transmittance 25%

Differentially accumulated metabolites (DAM) (CK vs -Q, CK vs -F, CK vs -H) between pair of samples were determined based on the variable importance in projection (VIP) ≥ 1 and fold change ≤0.5 or fold change ≥2 [32]. Our studies showed significantly high metabolites were differentially accumulated between the compared samples, including 40, 41, 40 DAMs in CK-vs-Q, CK-vs-F, CK-vs-H, respectively (Table S2). The KEGG top enriched among the DAMs were detected from all the compared samples for biosynthesis of secondary metabolites, anthocyanin biosynthesis, metabolic pathways, and flavonoid biosynthesis (Fig. 2A-C). Comparative analysis of the three groups of DAMs such as CK-vs-H、CK-vs-F and CK-vs-Q samples resolved to 37 common metabolites (Fig. 2D). The 37 metabolites including’s 4 down-accumulated and 33 up-accumulated compounds were constantly conserved the same patterns of differential accumulation (up- or down-) and may contain potential metabolites associated with peel coloration in blueberry (Table S3). We concluded that the DAMs from the flavonoid biosynthesis pathway were likely to be the key metabolites underlying the change in peel coloration of blueberry during natural light.

**Fig. 3:**
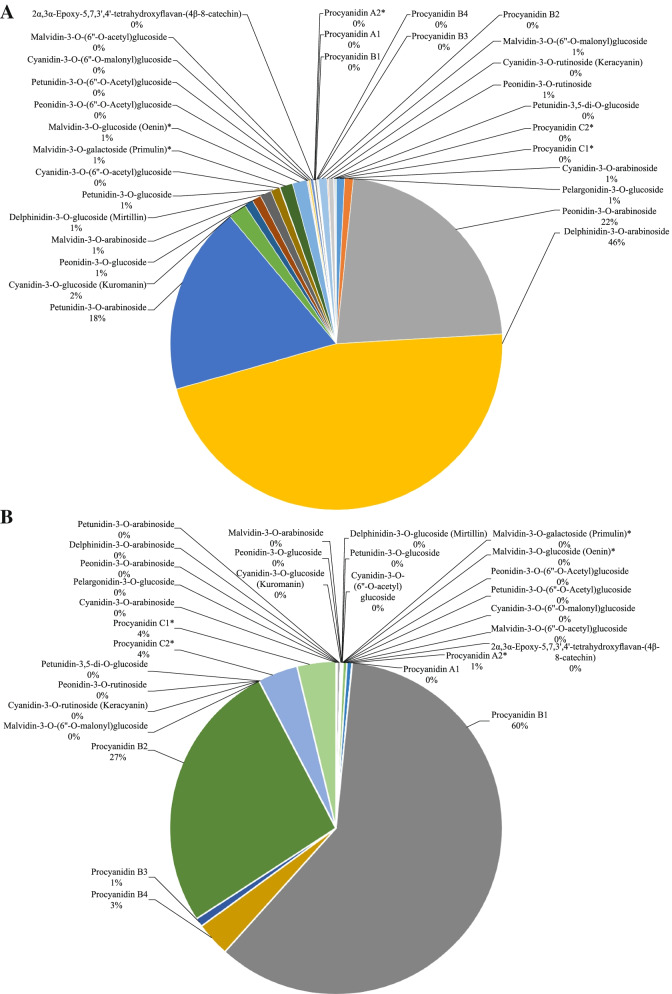
The proportion of individual anthocyanins in total anthocyanin concentration from CK (panel **A**) and Q (panel **B**) treatments

A total of 43 anthocyanins were detected in blueberries, 30 and 13 in CK and Q, respectively (Table S1). Analysis of the ratio of anthocyanin monomer content to total anthocyanin content in fruit: Delphinidin-3-O-arabinoside, Peonidin-3-O-arabino-side, and Petunidin-3-O-arabinoside accounted for total anthocyanin at 46, 22, and 18%, respectively, in CK, while it was 0 in Q (Fig. 3A). Procyanidin B1 and Procyanidin B2 accounted for 60 and 27%, respectively, in Q, and 0 in CK (Fig. 3B). This indicates that Delphinidin-3-O-arabinoside, Peonidin-3-O-arabino-side, Petunidin-3-O-arabinoside are light-inducible anthocyanins, and Delphinidin-3-O-arabinoside are light-inducible anthocyanins typical representatives of glycosides.

### Transcriptome analysis

**Fig. 4:**
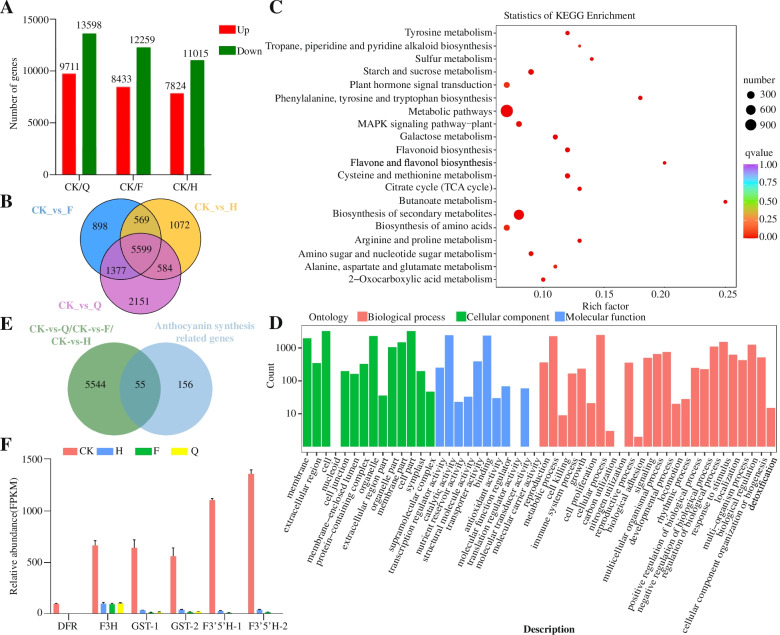
Number of differentially expressed genes (DEGs) identified by RNA-sequence analysis. **A** Numbers of DEGs. **B** Venn diagram representing numbers of DEGs. **C** Differential gene KEGG enrichment analysis. **D** Differential gene GO enrichment analysis. **E** Overlapping DEGs between 5599 DEGs and Genes related to anthocyanin synthesis pathway. **F** Transcript levels of overlapping DEGs between 5599 DEGs and Genes related to anthocyanin synthesis pathway as determined by RNA-seq

We obtained 81.57 G clean bases and 212 million clean reads from RNA sequence. As per sample, 53 million mean number of clean reads were calculated. From the clean reads, total mapped reads were 87.80, and 55.83% were mapped uniquely against the improved blueberry reference genome sequence (Table S4). We identified 29,539 DEGs in the three comparison groups. There were 23,308 DEGs in CK vs Q, 20702 in CK vs F, and 18,838 in CK vs H (Table S5). In the comparison groups, 9711, 8433, and 7824 genes were unregulated, and 13,598, 12,259, 11,015 genes were down-regulated (Fig. 4A). Generally, 5599 DEGs were up regulated from all three comparison groups (Fig. 4B).

The 5599 DEGs were subjected to GO and KEGG functional pathway analyses (Table S6). DEGs were categorized into DNA binding, plant hormone signal transduction, Biosynthesis of secondary metabolites, metabolic pathways, phenylalanine, tyrosine and tryptophan biosynthesis, and flavonoid biosynthesis (Fig. 4C, D). We obtained 5599 DEGs from anthocyanin synthesis pathway and screened 55 genes (Fig. 4E). Among these 55 genes, 6 genes are expressed in high levels in CK, but low in H, F. however, Q treatment was not expressed. These 6 DEGs encoded a Dihydroflavonol 4-reductase (K13082), two Glutathione transferases (K00799), a F3H(K00475), two Flavonoid-3′,5′-hydroxylases (K13083). Among them, *VcF3′5’H-2* (*VcF3′5’H4*) was the most up-regulated gene in the all treatment (Fig. 4F).

### Correlation analysis between selected transcripts and metabolites

**Fig. 5:**
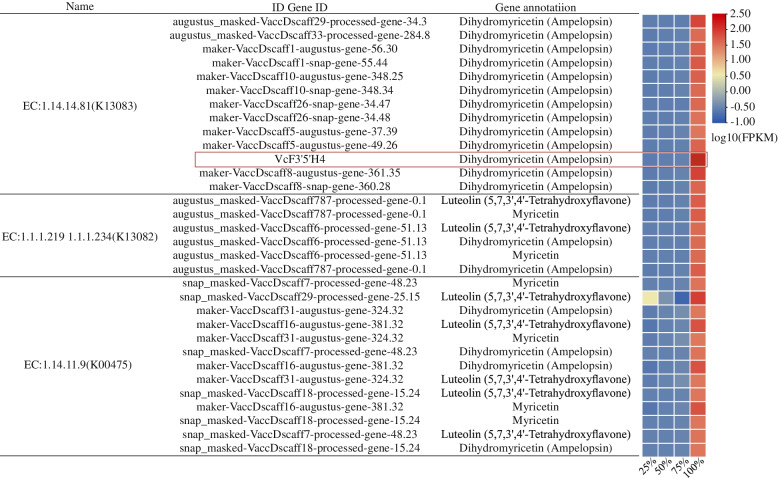
The results of the combined metabolome analysis of blueberry fruit transcription. Expression changes (right) and annotation (left) of the unigenes involved in flavonoid biosynthesis and phenyl-propanoid metabolism pathways in blueberry fruit. The gene ID and annotation indicates the gene product is related to the flavonoid biosynthetic pathway

The DEGs encoded by key enzymes in the anthocyanin synthesis pathway, such as EC: 1.14.14.81 (flavonoid 3’5’ hydroxylase, F3’5’H), EC: 1.14.11.9 (flavonoid 3-hydroxylase, F3H), and EC: 1.1.1.219 (dihydroflavonol 4-reductase, DFR) that are related to the production of metabolites and catalyze benzene the synthesis of flavonoids and anthocyanin glycosides in the propionic acid synthetic pathway (Fig. 5). The DEGs encoded by flavonoid 3’5’ hydroxylase catalyzed the production of dihydromyricetin. However, the DEGs encoded by dihydroflavonol 4-reductase DFR catalyzed the production of myricetin and dihydromyricetin. These DEGs were up-regulated under natural light, which was consistent with the accumulation pattern of blueberry fruit metabolites under natural light. These results indicated that the key enzyme genes and metabolites of the anthocyanin synthesis pathway were jointly involved in the formation of blueberry fruit taste, color and nutritional quality.

### Co-expression network analysis of genes related to anthocyanin synthesis

**Fig. 6:**
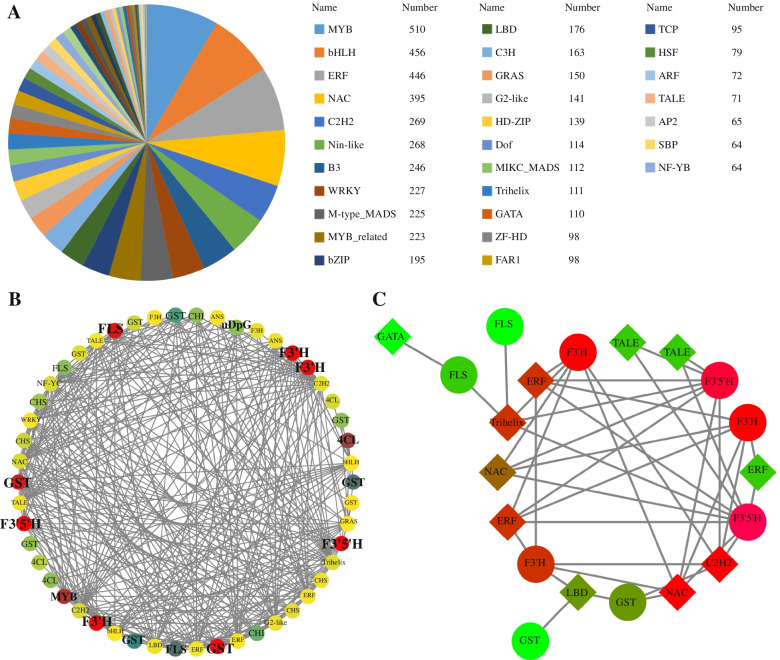
Transcription factor types and transcription factor co-expression network diagram. **A** Types of transcription factors, **B** Co-expression network diagram, *R* ≥ 0.9, **C** Co-expression network diagram, *R* ≥ 0.99

This study used online data from PlantTFDB to identify all the transcription factors in the blueberry genome. We determined a total of 6004 transcription factors in blueberries, belonging to 58 transcription factor (TF) families in which the first 5 families such as MYB (10), bHLH (456 entries), ERF (446 entries), NAC (395 entries) and C2H2 (269 entries) (Fig. 6A). In the anthocyanin synthesis pathway, co-expression network analysis with transcription factors. We found the transcription factors such as *VcbHLH004*, *VcERF061*, *VcNAC072* and the key gene *VcF3’5’H4* of the anthocyanin synthesis pathway were co-expressed, and the correlation coefficient was high (R ≥ 0.9) (Fig. 6B, C).

Correlation results showed that the expression levels of the three genes(*VcbHLH004*, *VcERF061* and *VcNAC072*) were all positively correlated with the expression levels of the structural gene *VcF3’5’H4*, which were 0.966, 0.996, and 0.997, respectively. The above research results indicate that these three regulatory factors may be involved in the synthesis and regulation of blueberry fruit anthocyanin under light induction.

### qPCR analysis of DEGs related to anthocyanin accumulation

**Fig. 7:**
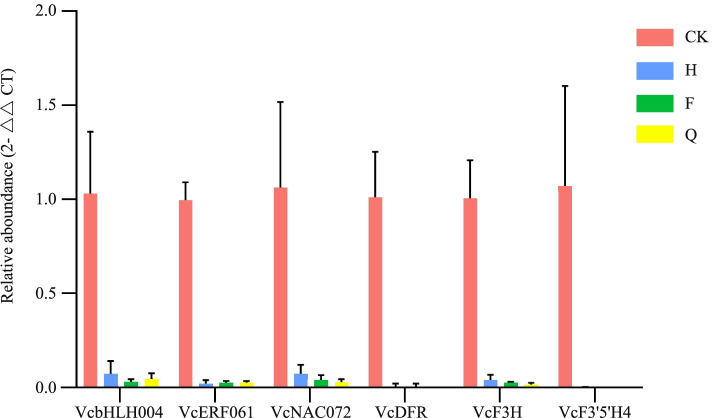
Transcript levels of anthocyanin related genes. Each experiment had three biological replicates

qPCR were conducted to analyze the 6 anthocyanin candidate genes such as *VcbHLH004*, *VcERF061*, *VcNAC072*, *VcF3H*, *VcDFR*, and *VcF3’5’H4* (for gene IDs and primers, see Table S8). The transcript profiles of all selected genes were highly similar to those detected from the RNA-sequence data (Fig. 7). The results showed that *VcF3’5’H4* was most up-regulated in different light intensity. This result was highly consistent with the results of RNA-sequence, and provided further evidence for the crucial role of *VcF3’5’H4* in anthocyanin accumulation in blueberry.

### *VcbHLH004*-mediated anthocyanin in blueberry

Co-expression network analysis showed that *VcbHLH004*, *VcF3’5’H4*, and *VcbHLH004* had the highest correlation with anthocyanin content (R2 = 0.897) (Table S9). Our combined metabolite and transcriptomic analyses revealed a core set of genes closely correlated with blueberry anthocyanin, which strongly suggested that they play key roles in anthocyanin accumulation in blueberries.

### Functional analysis of *VcbHLH004*

**Fig. 8:**
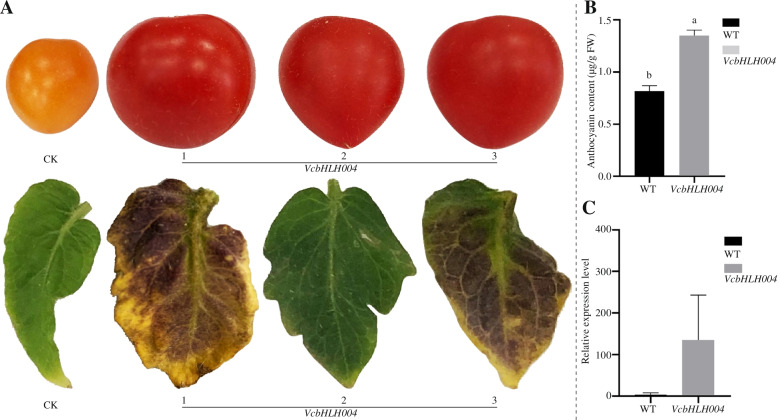
Phenotype analysis, anthocyanin content and target gene expression analysis of transgenic tomato plants. **A** The phenotype of transgenic leaf color and fruit color. **B** The anthocyanin content. **C** The expression of the target gene

The leaf and fruit colors of the *VcbHLH004* transgenic tomato plants were significantly darker than the WT control plant (Fig. 8A). The anthocyanin contents were also significantly increased (Fig. 8B). The results of the relative expression of the target gene showed that the relative expression of *VcbHLH004* in the fruits of the transgenic tomato plants increased significantly compared with the control plants (Fig. 8C). Our results indicated that *VcbHLH004* was a positive regulator of blueberry anthocyanin synthesis and accumulation by light.

## Discussion

### Effects of light intensity on flavonoids in blueberry

Flavonoids are a large group of plant-derived compounds that have tricyclic phenyl benzopyridine structure. It can be divided into anthocyanin glycosides, flavonoids, flavanols, flavonols, and isoflavones [33]. Flavonoids have the functions of resisting ultraviolet radiation, attracting insects to pollinate, and improving fruit quality. In our experiments, we identified 134 species of flavonoids using ultra-high performance liquid chromatography and tandem mass spectrometry technology. Flavonoids, of which 37 metabolites are common (Fig. 2). Anthocyanin, flavonols and their derivatives have been reported in recent years. In our study, these DAMs also found that were significantly accumulated in different light intensities. These DAMs are phenolic compounds that have antioxidant properties and are abundant in the pulp, juice, seed and peel of blueberry fruit [14]. The relative content of these DAM detected with the increase of light intensity in different light environments, especially reaching the highest level under natural light. The expression levels of genes related to DAM biosynthesis determined by RNA-seq and qRT-PCR had a similar tendency to the metabolome profile. These results indicate that the DAMs and related genes identification were important factors affecting blueberry fruit color during different light intensity.

Anthocyanins are the most important substances affect the fruit color of plants. Various studies have shown that the accumulation of anthocyanins are affected by environmental factors, especially light intensity [34]. In grape barriers, anthocyanin accumulation increases due to strong light, while shading inhibits [35]. Strong light inhibits the accumulation of anthocyanin in *petunia atkinsiana* [36]. Our results showed that with the increase of light intensity, the accumulation of anthocyanin increased and the color of the fruit become dark (Fig. 1), which is consistent with the results of Li et al. on Nangaocong ‘O’Neal’ [14]. Strong light promotes blueberry anthocyanin accumulation. This type of proportion, distribution and interaction of chlorophyll, flavonoids (anthocyanins, flavonoids), carotene and other pigments determine the color of the peel [37]. Our research showed that as the light intensity increases, the content of total flavonoids, chlorophyll and carotenoids decreases which is consistent with the result of Nguyen et al. [37]. These results showed that the coloring of blueberry fruit is mainly due to the accumulation of anthocyanins.

### Light-induced anthocyanin components can be divided into two categories

We identified 40, 41, and 40 differentially accumulated metabolites in CK-vs-Q, CK-vs-F and CK-vs-H, respectively. Comparative analysis of the three groups of DAMs such as CK-vs-H、CK-vs-F and CK-vs-Q samples resolved to 37 common metabolites. The KEGG pathway analysis results for the differentially accumulated metabolites showed similar results. The flavonoid biosynthesis for intermediate product accumulation were significantly enriched in CK-vs-Q, CK-vs-F, CK-vs-H. Our findings are similar to those of Ma et al. (2019) and this indicates that the metabolites in the flavonoid synthesis pathway may be the key metabolites for blueberry fruit accumulation under light.

The GO analysis results showed that most of the DEGs were classified into functional categories such as, Cellular Component (CC), and Molecular Function (MF)” category. These GO terms indicate light-induced metabolic processes that occur during fruit ripening formation. We have previously shown that the gene expression patterns of *VcCOP1*, *VcUFGT*, *VcF3’5’H* and *VcHY5* are responsive to light after shading net removal in a light-sensitive blueberry variety. Additionally, the expression of the anthocyanin-related biosynthesis genes *PyDFR*, *PyANS*, and *PyUFGT* and the transcription factor genes *PyMYB10*, *PybHLH33*, and *PyWD40* showed a positive correlation with light-induced anthocyanin accumulation [38].

The composition of anthocyanins in the fruit affects the coloration of the fruit. At the same time, the results of this study showed that the composition of anthocyanins gradually decreased with the decrease of light transmittance. 30 and 13 anthocyanins were detected in CK and Q, respectively. Delphinidin-3-O-arabinoside, Peonidin-3-O-arabinoside, and Petunidin-3-O-arabinoside were only detected in CK. Delphinidin-3-O-arabinoside O-arabinoside accumulated a lot, while Procyanidin B1 and Procyanidin B2 were only detected in Q, indicating that Delphinidin-3-O-arabinoside is a typical representative of light-inducible anthocyanins. The effect of light on fruit has been reported in previous studies, such as shading reduced the accumulation of total anthocyanins and 3′-hydroxylated anthocyanins in ‘Nebbiolo’ grape fruit, but increased the 3′, 5′- The concentration of hydroxylated anthocyanins [39]. Likewise, the content of 3, 4′, 5′-hydroxylated anthocyanins was reduced in ‘Yan-73’ under dark conditions [40].

A photosensitive *VcbHLH004* gene plays an important role during light-induced anthocyanin biosynthesis. Anthocyanin and flavonoid synthesis are regulated by several structural genes and TFs such as MYB, bHLH and WDR proteins. Karppinen et al. (2021) showed that *VmMYBPA1.1* can activate *F3’ 5’H, DFR*, *ANS* and *UFGT* promoter activities, thereby promoting blueberry anthocyanin accumulation [41]. The bHLH proteins can interact with R2R3-MYBs from various subgroups, and form ternary complexes with WDR. The MBW (MYB-bHLH-WDR) complexes participated in flavonols, anthocyanin biosynthesis pathway [42, 43]. The bHLH played an important role in anthocyanin synthesis by forming a complex with MYBs. The first bHLH transcription factor was discovered in maize, where its function is involved in the synthesis of anthocyanin [44]. In apples, the *MdbHLH3* transcription factor has been shown to promote anthocyanin accumulation in fruits [45]. Overexpression of *SlPRE2*, an atypical bHLH, accelerated seedling morphogenesis and produced yellowing ripen fruits with reduced chlorophyll and carotenoid in tomato fruit [46]. Overexpression of *SlPRE2*, an atypical bHLH accelerated seedling morphogenesis and produced yellowing ripen fruits with reduced chlorophyll and carotenoid in tomato fruit [47]. Wang et al. showed that 11 bHLHs were up-regulated in Lv fruit skin, while seven bHLHs were significantly down regulated compared with Bai suggested that bHLHs function as different roles in biosynthesis of anthocyanin [48]. Li et al. believed that the light-induced bHLH transcription factor *FvbHLH9* is a positive regulator of anthocyanin synthesis, and *FvHY5* specifically binds to the promoter regions of some key enzyme genes including FvDFR, and the expression of *FvDFR* [49]. It is activated by the formation of a heterodimer between *FvHY5* and *FvbHLH9*, thereby promoting the accumulation of strawberry anthocyanin [49]. Ma et al. used Marselan’ grape as material to conduct transient expression experiments and found that after overexpression of *VvMYB30*, *VvbHLH79* and *VvbHLH121*, the content of anthocyanin at the injection site and the expression of the target gene were significantly increased compared with the control [16]. Therefore, it is believed that *VvMYB30*, *VvbHLH79* and *VvbHLH121* positively regulate the synthesis and accumulation of glucosinolates. In this study, overexpression of *VcbHLH004* in tomato can accelerate fruit morphogenesis, darken leaves, reddish fruits, increased anthocyanin content, and up-regulation of target genes. This indicates that *VcbHLH004* positively regulates light-induced anthocyanin synthesis in blueberry.

Multiomics analysis reveals new links between transcription and metabolism. Correlation analysis between the transcriptome and metabolome reveals differentially accumulated metabolites that are related to phenotypic change and the DEGs that cause the changes in metabolites.

**Fig. 9:**
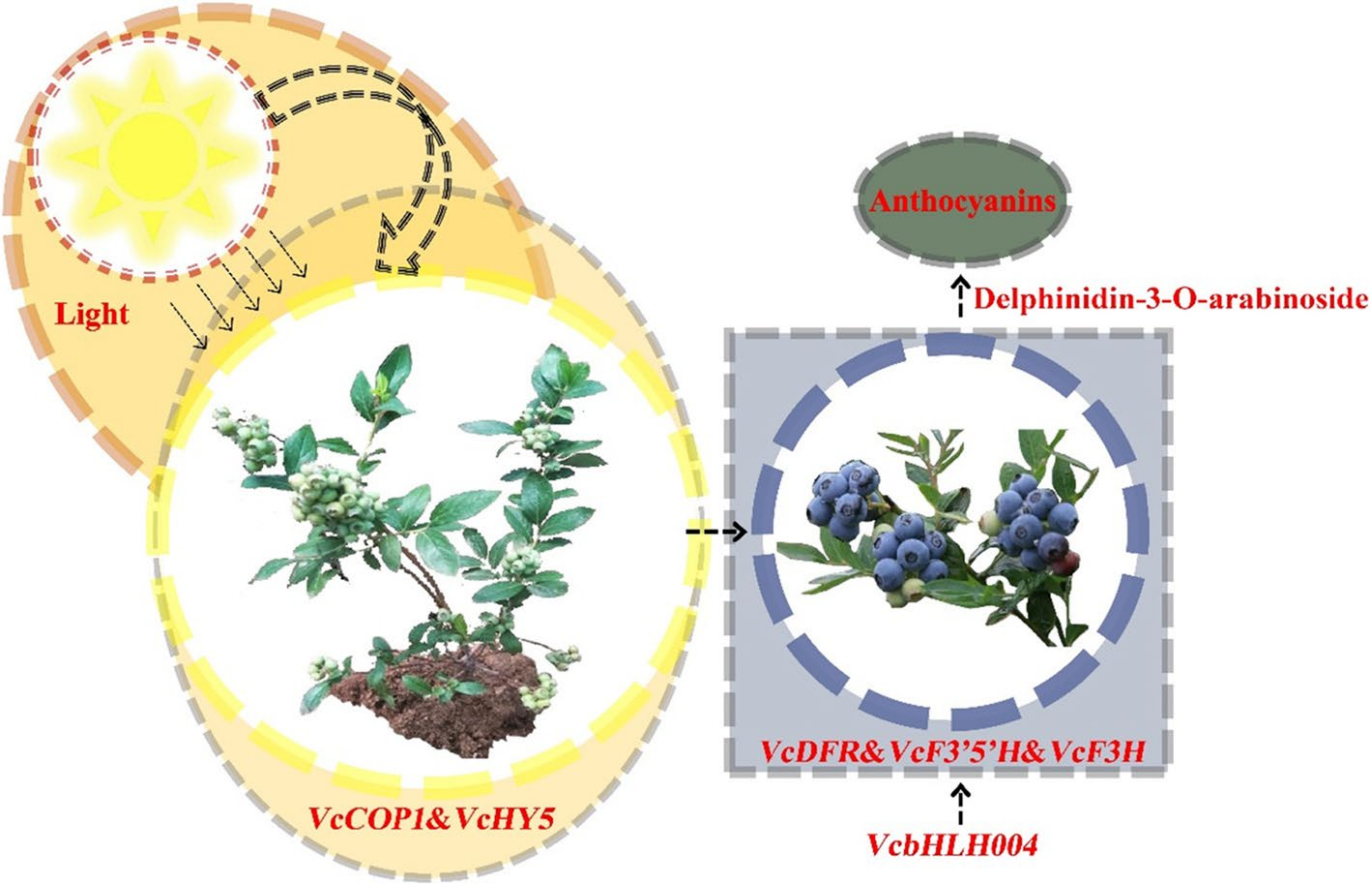
Light-induced anthocyanin biosynthesis regulation model in blueberry

This integrative analysis approach makes it easier to identify key regulatory metabolic pathways and reliable key regulatory genes [50, 51]. Combining omics analysis of diverse genetic resources provides crucial information in understanding molecular basis of plant fruit coloring, such wild peach species in flesh coloration [52], *Ziziphus jujuba* Mill fruit color [53], and cucumber fruit skin color [48]. Herein, we used RNA-seq and metabolomics to characterized four different blueberry on fruit skin color (CK, H, F, Q) using RNA-seq and metabolome. CK is dark purple with high anthocyanin content. We also analyzed the different metabolites, flavones, flavanones, flavonols, and anthocyanins, were mostly responsible for skin color differences (Fig. 2). In addition, combining transcript level by RNA-seq, we found that several DEGs related to anthocyanins synthesis and TFs were possibly involved in the color development. The metabolome data combining with transcriptome profiling were discovered genes involved in anthocyanins synthesis, thus searching for useful information to illustrate phenomenon of different color in blueberry fruit. Anthocyanins are the final products of the flavonoid biosynthetic pathways. Our study showed many DEGs are differently expressed between Q、F、H and CK in this pathway, such as upstream *VcDFR*, *VcF3H* and *VcF3’5’H4*. Previous studies have shown that *F3’5’H* gene plays an important role in light-induced anthocyanin biosynthesis [20]. Ma et al. (2019) found that the content of *F3’5’H* (VIT_06s0009g02840) and Malvidin-3-O-coumaroylglucoside (cis) was significant positively correlated under different light intensities in grapes (*V. vinifera* L.), and speculated it may be a positive regulator of anthocyanin synthesis [16]. The *F3’5’H* expression was associated with anthocyanin accumulation in different plant. Our results showed that *VcF3’5’H4* expressions are suppressed in Q treatment, it maybe explain three types of anthocyanin down-regulation in Q treatment as compared to CK. This indicated that *VcF3’5’H4* may be the key gene for fruit anthocyanin accumulation under natural light. Other researcher found that the Delphinidin-3-O-arabinoside played an important role in skin of ‘O’Neill’in Blueberry [14]. Using this method, we also identified a light-responsive transcription factor, *VcbHLH004*, which may promote anthocyanin accumulation by binding to the *VcF3’5’H4* promoter. Based on this and previous studies, we propose a regulatory network for anthocyanin biosynthesis in blueberry (Fig. 9). Our results show the significance of integrated multiomics approaches for understanding plant physiological processes and provide a case study of the analysis of molecular mechanisms based on multiomics.

## Conclusion

In this study, we analyzed the metabolome and transcriptome of blueberry fruits under different light intensities. Using an integrated analysis of the differential metabolites and genes expression levels, we identified the metabolites involved in responses to different light intensities. We further analyzed the differential expression of genes involved in anthocyanin biosynthesis using qRT-PCR. Through the analysis of transcriptome data and metabolome data, it is found that the *VcF3’5’H4* gene may play an important role in the light-induced blueberry anthocyanin synthesis pathway. Through the co-expression analysis of transcription factors and anthocyanin synthesis pathway genes, we found that *VcbHLH004* gene may regulate *VcF3’5’H4*, transformed *VcbHLH004* heterologous into tomato to verify its function. The candidate genes for blueberry anthocyanin accumulation presented here represent a valuable data set to guide future functional studies.

## Supplementary Information

**Additional file 1: Figure S1.** Heat map analysis of compound composition under different light intensity. **Table S1.** Compound classification. **Table S2.** Differential metabolite analysis. **Table S3.** 37 common metabolites. **Table S4.** Overview of mapping of RNA-seq reads. **Table S5.** Differential gene analysis. **Table S6.** Annotation analysis of differential genes KEGG and GO. **Table S7.** Screening genes for anthocyanin synthesis pathway. **Table S8.** qRT-PCR. **Table S9.** Correlation analysis between anthocyanin content and key genes.

## Data Availability

The raw data presented in this study are available on request from the corresponding author. The data are not yet publicly available since the project is still ongoing.
