# Supplementary material for: Metabolome and transcriptome profiling unveil the mechanisms of light-induced anthocyanin synthesis in rabbiteye blueberry (vaccinium ashei: Reade)
Source: BMC Plant Biol. 2022 Apr 29;22:223. doi: 10.1186/s12870-022-03585-x (PMC9052483; doi:10.1186/s12870-022-03585-x)
Supplement: Supplementary file 1 — Additional file 1: Figure S1. Heat map analysis of compound composition under different light intensity. Table S1. Compound classification. Table S2. Differential metabolite analysis. Table S3. 37 common metabolites. Table S4. Overview of mapping of RNA-seq reads. Table S5. Differential gene analysis. Table S6. Annotation analysis of differential genes KEGG and GO. Table S7. Screening genes for anthocyanin synthesis pathway. Table S8. qRT-PCR. Table S9. Correlation analysis between anthocyanin content and key genes. [file 12870_2022_3585_MOESM1_ESM.zip › TableS9.docx]

|  | Anthocyanin | *VcDFR* | *VcF3H* | *VcF3'5'H4* | *VcNAC072* | *VcbHLH04* | *VcERF061* |
| --- | --- | --- | --- | --- | --- | --- | --- |
| Anthocyanin | 1 |  |  |  |  |  |  |
| *VcDFR* | 0.770** | 1 |  |  |  |  |  |
| *VcDFR* | 0.761** | 0.995** | 1 |  |  |  |  |
| *VcF3'5'H4* | 0.780** | 0.998** | 0.996** | 1 |  |  |  |
| *VcNAC072* | 0.814** | 0.996** | 0.993** | 0.997** | 1 |  |  |
| *VcbHLH04* | 0.897** | 0.966** | 0.958** | 0.971** | 0.983** | 1 |  |
| *VcERF061* | 0.809** | 0.997** | 0.991** | 0.997** | 0.999** | 0.981** | 1 |

Table S9: Correlation analysis between anthocyanin content and key genes
